# The *Aspergillus fumigatus* Mucin MsbA Regulates the Cell Wall Integrity Pathway and Controls Recognition of the Fungus by the Immune System

**DOI:** 10.1128/mSphere.00350-19

**Published:** 2019-06-19

**Authors:** Isabella Luísa da Silva Gurgel, Karina Talita de Oliveira Santana Jorge, Nathália Luísa Sousa de Oliveira Malacco, Jéssica Amanda Marques Souza, Marina Campos Rocha, Marina Faria Fernandes, Flávia Rayssa Braga Martins, Iran Malavazi, Mauro Martins Teixeira, Frederico Marianetti Soriani

**Affiliations:** a Centro de Pesquisa e Desenvolvimento de Fármacos, Instituto de Ciências Biológicas, Departamento de Genética, Ecologia e Evolução, Universidade Federal de Minas Gerais, Belo Horizonte, Brazil; b Departamento de Genética e Evolução, Centro de Ciências Biológicas e da Saúde, Universidade Federal de São Carlos, São Carlos, São Paulo, Brazil; c Centro de Pesquisa e Desenvolvimento de Fármacos, Departamento de Bioquímica e Imunologia, Instituto de Ciências Biológicas, Universidade Federal de Minas Gerais, Belo Horizonte, Brazil; Carnegie Mellon University

**Keywords:** *Aspergillus fumigatus*, cell wall integrity, immune response, *msb2*, mucin, virulence

## Abstract

Aspergillus fumigatus is a filamentous fungus which causes invasive pulmonary aspergillosis in immunocompromised individuals. In fungi, cell signaling and cell wall plasticity are crucial for maintaining physiologic processes. In this context, Msb2 is an important signaling mucin responsible for activation of a variety of mitogen-activated protein kinase (MAPK)-dependent signaling pathways that regulate cell growth in several organisms, such as the cell wall integrity (CWI) pathway. Here, we aimed to characterize the MSB2 homologue in A. fumigatus. Our results showed that MsbA plays a role in the vegetative and reproductive development of the fungus, in stress adaptation, and in resistance to antifungal drugs by modulating the CWI pathway gene expression. Importantly, cell wall composition is also responsible for activation of diverse receptors of the host immune system, thus leading to a proper immune response. In a model of acute *Aspergillus* pulmonary infection, results demonstrate that the Δ*msbA* mutant strain induced less inflammation with diminished cell influx into the lungs and lower cytokine production, culminating in increased lethality rate. These results characterize for the first time the role of the signaling mucin MsbA in the pathogen A. fumigatus, as a core sensor for cell wall morphogenesis and an important regulator of virulence.

**IMPORTANCE** Aspergillus fumigatus is an opportunistic fungus with great medical importance. During infection, *Aspergillus* grows, forming hyphae that colonize the lung tissue and invade and spread over the mammal host, resulting in high mortality rates. The knowledge of the mechanisms responsible for regulation of fungal growth and virulence comprises an important point to better understand fungal physiology and host-pathogen interactions. Msb2 is a mucin that acts as a sensor and an upstream regulator of the MAPK pathway responsible for fungal development in Candida albicans and Aspergillus nidulans. Here, we show the role of the signaling mucin MsbA in the pathogen A. fumigatus, as a core sensor for cell wall morphogenesis, fungal growth, and virulence. Moreover, we show that cell wall composition, controlled by MsbA, is detrimental for fungal recognition and clearance by immune cells. Our findings are important for the understanding of how fungal sensors modulate cell physiology.

## Abstract (precis)

Aspergillus fumigatus is an opportunistic fungus with great medical importance. During infection, *Aspergillus* grows, forming hyphae that colonize the lung tissue and invade and spread over the mammal host, resulting in high mortality rates. The knowledge of the mechanisms responsible for regulation of fungal growth and virulence comprises an important point to better understand fungal physiology and host-pathogen interactions. Msb2 is a mucin that acts as a sensor and an upstream regulator of the MAPK pathway responsible for fungal development in Candida albicans and Aspergillus nidulans. Here, we show the role of the signaling mucin MsbA in the pathogen A. fumigatus, as a core sensor for cell wall morphogenesis, fungal growth, and virulence. Moreover, we show that cell wall composition, controlled by MsbA, is detrimental for fungal recognition and clearance by immune cells. Our findings are important for the understanding of how fungal sensors modulate cell physiology.

## INTRODUCTION

Fungal cell survival is dependent on the organization, composition, and function of the cell wall in which synthesis and remodeling are highly regulated. The cell wall integrity (CWI) pathway is responsible for the maintenance of a rigid but dynamic cell wall, which is important for hyphal growth, adaptation to environmental challenges, and host invasion and colonization (1). The CWI functionality relies on the activation of external sensors and the downstream activation of the mitogen-activated protein kinase (MAPK) cascade, which in turn phosphorylates targeted transcription factors that ultimately induce the expression of different proteins involved in cell wall reinforcement (1–3). In addition, external sensors, such as mucins, are able to sense the activity of O-mannosyltransferases and the protein glycosylation status via its extracellular domain. As a result, the cleaved cytoplasmic domain can mobilize Cdc42, which is essential for MAPK activation and cell wall rearrangement (4, 5).

Signaling mucins are transmembrane proteins that play an important role in cell wall plasticity after O-glycosylation. Members of this class are integral-membrane glycophosphatidylinositol (GPI)-anchored proteins with a cytoplasmic domain, which interfaces with signaling transduction machinery (5). The signaling mucin Msb2 activates the CEK1-MAPK pathway in Candida albicans, KSS1 in Saccharomyces cerevisiae, Fmk1 in Fusarium oxysporum, and the cell wall integrity pathway in Aspergillus nidulans. Msb2 signaling has also been extensively characterized as an external sensor for cell growth (4, 6–12).

Msb2 has also been recognized as a virulence determinant in pathogenic fungi, such as C. albicans. Indeed, Szafranski-Schneider et al. (13) demonstrated that upon release to the outer cell space, the glycosylated extracellular domain of Msb2 protects the fungal cells from antimicrobial peptides such as histatin and cathelicidins produced by the mucosa and immune cells of the host such as neutrophils and macrophages (13–16). Also, Msb2 is responsible for activation of the MAPK Fmk1 in the soilborne fungus F. oxysporum, regulating invasive growth and virulence (7). Additionally, in the rice blast fungus Magnaporthe oryzae, Msb2 is responsible for appressorium formation, penetration, and invasive growth through activation of the Pmk1 MAPK pathway and Ras2 GTPase (10).

In the present study, we have identified and characterized an S. cerevisiae *MSB2* homologue, in the pathogenic filamentous fungus A. fumigatus, named here *msbA*. We show that *msbA* influences vegetative and reproductive growth, reflecting into the cell-cell adhesion properties and biofilm formation. These phenotypes are related to a modulation of the expression of the CWI pathway genes that are thought to control cell wall composition. MsbA is shown to be crucial for survival in an immunocompetent model of A. fumigatus lung infection. Moreover, an *msbA* mutant modulates activation of the immune system affecting cell influx into the airways and production of inflammatory mediators. Altogether, we demonstrate for the first time that the A. fumigatus signaling mucin, MsbA, represents a core sensor for cell wall morphogenesis and an important regulator of virulence.

## RESULTS

### Identification of the Msb2 homologue in A. fumigatus and construction of the *ΔmsbA* mutant strain.

**FIG 1:**
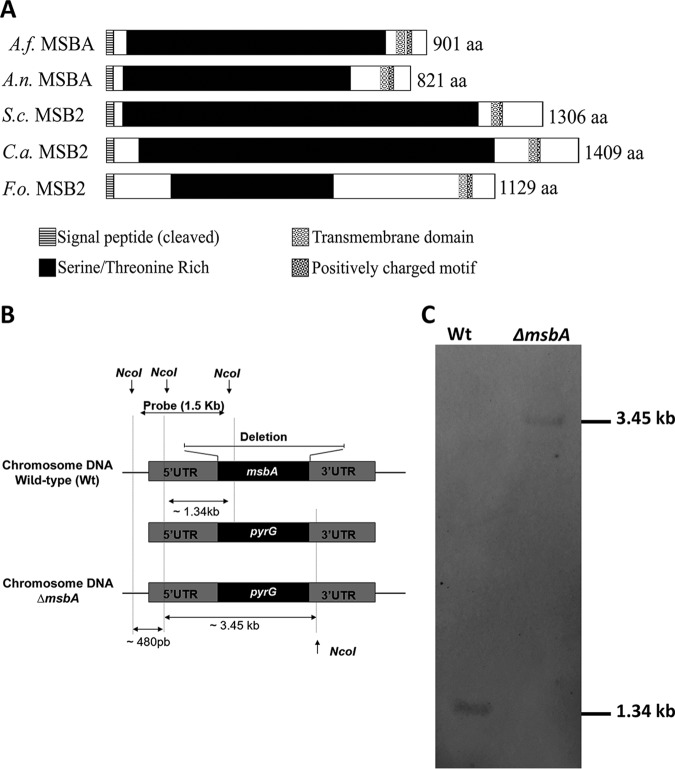
Domain architecture prediction and knockout strain construction. (A) A. fumigatus MsbA shares common features of signaling mucins with A. nidulans MSBA, S. cerevisiae MSB2, C. albicans MSB2, and F. oxysporum MSB2. All of these mucin proteins contain a cleaved signal peptide (SignalP) and one transmembrane domain (TMHMM) close to the C terminus. The large extracellular part is Ser/Thr rich (ProtParam; Color Protein Sequence). Right after the transmembrane region, the short cytoplasmic tail contains a positively charged motif (RR-RKKR--HRR in A. fumigatus; RR-RKKR--HRR in A. nidulans; RRR in S. cerevisiae; RK-RK in C. albicans; and RR-KRKK---HRR in F. oxysporum). (B) The *msbA* gene was replaced, through homologous recombination, by the auxotrophic marker *pyrG.* (C) Southern blotting of wild-type strain presenting a fragment of 1.34 kb and the knockout strain (*ΔmsbA*) presenting a fragment of 3.45 kb.

In order to identify the putative *MSB2* orthologue in A. fumigatus, we performed a BLASTp search using S. cerevisiae *MSB2* and A. nidulans *MSBA* as queries. Our search revealed a putative orthologue in A. fumigatus, Afu4g04070, named here *msbA* to be consistent with A. nidulans nomenclature. The A. fumigatus *msbA* gene is a 2,706-nucleotide open reading frame, located in the short arm of chromosome 4, with a predicted 901-amino-acid protein sequence. Comparisons of *msbA* with homologue sequences from other fungal species denote a high identity of 75.6% (E value 7.7e−61) with A. nidulans *MSBA* but 36.1% (E value 4.3e−5) protein identity with S. cerevisiae *MSB2,* 36.4% (E value 1.4e−4) with C. albicans *MSB2,* and 34.5% (E value 1.9e−17) with F. oxysporum *MSB2*. Moreover, *in silico* analysis demonstrated that A. fumigatus MsbA protein contains a single transmembrane region as well as serine/threonine-rich regions, both common features in other MSB2 mucin proteins previously described (Fig. 1A). In addition, it has been described that mucins undergo posttranslational modifications, especially glycosylation. We performed a prediction of O-GlcNAcylated sites and observed that A. fumigatus MsbA presents a similar distribution pattern of these sites as reported for other fungal mucins (data not shown).

To determine the role of *msbA* in A. fumigatus, a deletion mutant strain was constructed by replacing the genomic sequence of *msbA* with the *pyrG* marker in the *ΔakuB^Ku80^* strain (wild type) (17). The double crossover of the deletion cassette occurred in approximately 100 transformants, which were confirmed by Southern blotting (Fig. 1B). After genomic DNA restriction by NcoI, we were able to identify a 1.34-kb fragment in the wild-type strain and a fragment of approximately 3.45 kb in the Δ*msbA* mutant strain (Fig. 1C). We also constructed the complemented strain by reconstitution of the *msbA* gene. The complementation of the *msbA* gene in the *ΔmsbA* mutant background was confirmed by PCR (see Fig. S1A in the supplemental material).

10.1128/mSphere.00350-19.1 FIG S1 Complemented strains reverted the mutant phenotype. (A) Complemented strain construction confirmation by PCR. Amplification of the *msbA* gene was performed by PCR to confirm the reinsertion of the gene *msbA* in the *ΔmsbA* genome. In the first well, wild-type DNA was used as a control; second well represents the DNA ladder. The other lanes represent candidates for the complementation. Complementation candidates 1 and 2 were chosen for the experiments and named *ΔmsbA*::*ΔmsbA* 1 and *ΔmsbA*::*ΔmsbA* 2, respectively. (B to E) Complemented strains *ΔmsbA*::*ΔmsbA* 1 and *ΔmsbA*::*ΔmsbA* 2 reverted the growth phenotype. Radial growth was evaluated at 30°C, 37°C, and 42°C in YAG after 48 h. Quantification was performed by colony diameter measurement every 24 h for 96 h. * represents statistical difference (*P* < 0.05) from the mutant strain under the same growth condition. Number sign represents statistical difference (*P* < 0.05) from the wild type under the same growth condition. (F) Complemented strains reverted the cell wall stress sensitivity. Tenfold dilution dropout growth in solid MM plates supplemented with different cell wall-perturbing agents: Congo red (CR), calcofluor white (CFW), and NaCl. Download FIG S1, TIF file, 2.0 MB. Copyright © 2019 Gurgel et al. 2019 Gurgel et al. This content is distributed under the terms of the Creative Commons Attribution 4.0 International license.

### *msbA* is involved in the maintenance of vegetative growth and adhesion ability of A. fumigatus.

**FIG 2:**
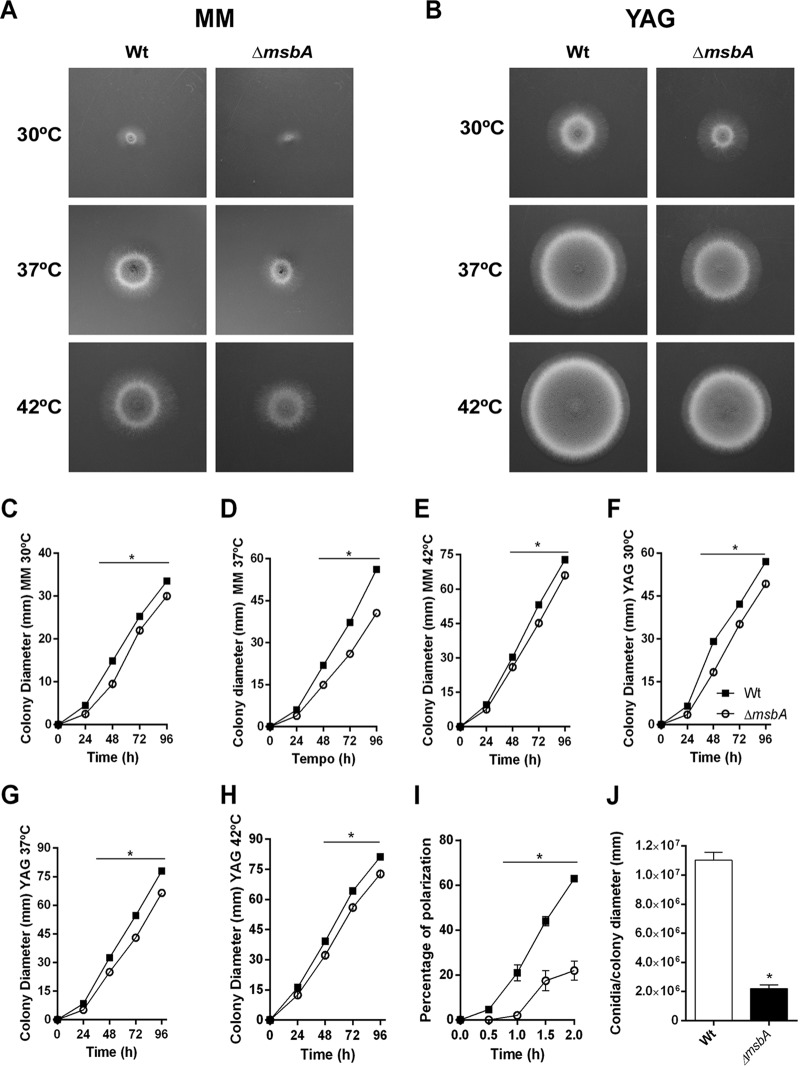
The Δ*msbA* strain has reduced vegetative growth and conidiation. The mutant strain has a diminished radial growth at 30°C, 37°C, and 42°C compared to wild type, in both MM and YAG. (A) MM for 48 h; (B) YAG for 48 h; (C to E) MM at 30°C, 37°C, and 42°C, respectively; (F to H) YAG at 30°C, 37°C, and 42°C, respectively. Quantification was performed by colony diameter measurement every 24 h during 96 h (C to H). Asterisk represents statistical difference (*P* < 0.05) from wild type under the same growth condition. (I) Percentage of polarized growth after HU release. Samples of germlings were analyzed at 0 h, 0.5 h, 1 h, 1.5 h, and 2 h after release of HU blockade. Asterisk represents statistical difference (*P* < 0.05) from wild type under the same growth condition. (J) Number of conidia/colony diameter after growth in MM at 37°C for 72 h. Asterisk represents statistical difference (*P* < 0.05) from wild type.

**FIG 3:**
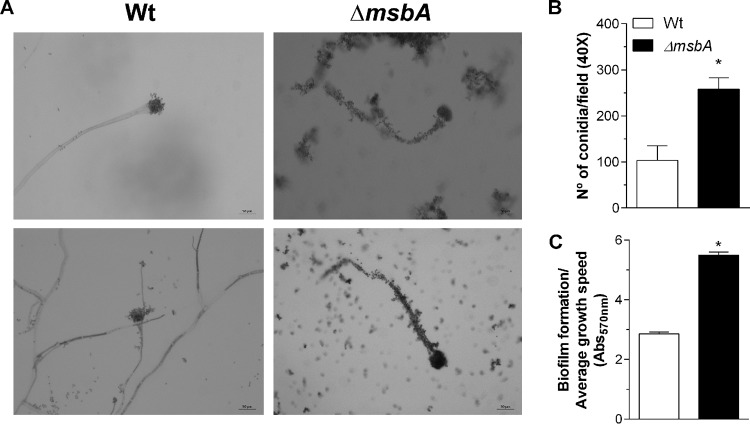
The Δ*msbA* strain shows enhanced adhesion properties and biofilm formation. (A) Optical microscopy of conidiophores from wild-type and Δ*msbA* strains. On the right side are shown two independent experiments on wild-type conidiophores. On the left side are shown two independent experiments on Δ*msbA* conidiophores. The coverslips were stained using lactophenol blue solution and analyzed by optical microscopy using ×40 magnification. Bars, 50 μm. Experiments were performed in triplicate. (B) Adhesion properties of conidia were analyzed after 4 h of growth in RPMI at 37°C. Adhered conidium number was determined in at least 6 microscope fields and expressed as total percent conidia in each assay. (C) Biofilm formation was measured indirectly by absorbance at 570 nm. Absorbance values were normalized by growth rate of each strain in MM. Values are shown as mean ± SEM. Asterisks represent statistical difference (*P* < 0.05) from wild type under the same growth condition.

In A. nidulans*, msbA* regulates filamentous growth and the CWI pathway (11); thus, we tried to understand the role played by *msbA* in the maintenance of cell wall integrity in A. fumigatus. Initially, we investigated the role of *msbA* in vegetative growth of A. fumigatus. Colonies lacking *msbA* had impaired radial growth at 30°C, 37°C, and 42°C on both minimal medium (MM) and complete medium (YAG) (Fig. 2A and B, respectively). The effect on radial growth was more evident at 37°C, the optimal temperature of growth, with a reduction of about 30% in colony diameter of the *ΔmsbA* mutant. The alteration of growth phenotype was quantified and is shown in Fig. 2C to H. The complemented strain reverted the defective growth phenotype of the mutant strain (Fig. S1B to E). Furthermore, conidium formation was also affected by deletion of *msbA,* which is evidenced by the decrease in the number of conidia produced by the *ΔmsbA* strain compared to the wild-type strain (Fig. 2J). We hypothesized that the morphotypic alteration in the conidiophores of the Δ*msbA* strain could explain the decrease of conidiation. However, microscopic analyses of the aerial structures of the null mutant showed no evident morphologic differences between strains (Fig. 3A).

We also observed that *msbA* deletion leads to a significant delay in germ tube formation in liquid medium. Only 20% of mutant strain conidia emitted germ tubes compared to approximately 65% of the wild-type conidia after 2 h of incubation (Fig. 2I). These results indicate that vegetative growth is controlled, at least in part, by *msbA* and that, *in* A. fumigatus, conidiation is affected by this mucin, independently of morphotypic alterations of conidiophores.

From the beginning of the initial phenotypic analyses, we observed that Δ*msbA* conidia massively adhered to elongated hyphae, which was not evident for the wild-type hyphae, suggesting an alteration in conidial adhesion properties. Therefore, we assessed the adherence ability of A. fumigatus mutant strain conidia. *ΔmsbA* conidium adhesion was increased about 2.5 times compared to the wild-type strain (Fig. 3B). Adhesion is the initial phase of biofilm formation. Consistently, biofilm formation was significantly higher in the *ΔmsbA* strain than the wild type after 24 h of incubation at 37°C (Fig. 3C).

### *msbA* plays a role in maintaining the cell wall integrity.

**FIG 4:**
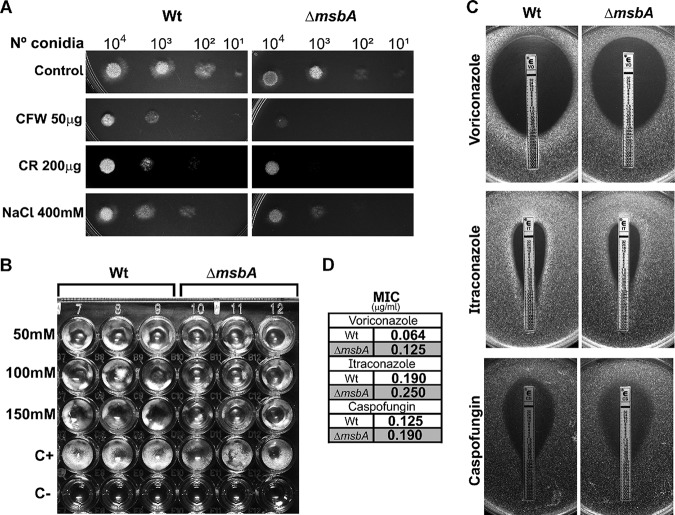
The *ΔmsbA* strain has altered sensitivity to cell wall stressors and antifungal agents. (A) Tenfold dilution dropout growth in solid MM plates supplemented with different cell wall-perturbing agents: Congo red (CR), calcofluor white (CFW), and NaCl. (B) Conidia (1 × 10^4^ of each strain) were incubated in liquid MM at 37°C for 48 h in the presence of the indicated concentrations of nikkomycin Z. (C) Antifungal susceptibility using Etest gradient strips for voriconazole, itraconazole, and caspofungin. (D) MICs of antifungal drugs analyzed in panel C.

Cell adhesion and biofilm formation are intimately related to cell wall properties. We subsequently evaluated the sensitivity of *ΔmsbA* to a variety of cell wall stressors. The *ΔmsbA* mutant strain presented increased sensitivity to chitin binding and chitin synthesis inhibitor agents, such as Congo red (CR), calcofluor white (CFW), and nikkomycin Z (Fig. 4A and B). It has also been demonstrated that Msb2 is able to sense osmolarity changes in the high-osmolarity glycerol (HOG) pathway in S. cerevisiae (18). Likewise, MsbA also seems to play a role in osmosensing in our system, as the mutant strain presented higher sensitivity to the osmotic stressor agent NaCl (Fig. 4A). The complemented strain reverted these phenotypes (Fig. S1F).

Moreover, we assessed the sensitivity of the mutant strain in the presence of different antifungal drugs. An Etest assay demonstrated that the *ΔmsbA* strain shows higher MICs of voriconazole and itraconazole (inhibitors of ergosterol biosynthesis) and caspofungin (β-1,3-glucan synthase inhibitor) (Fig. 4C and D).

**FIG 5:**
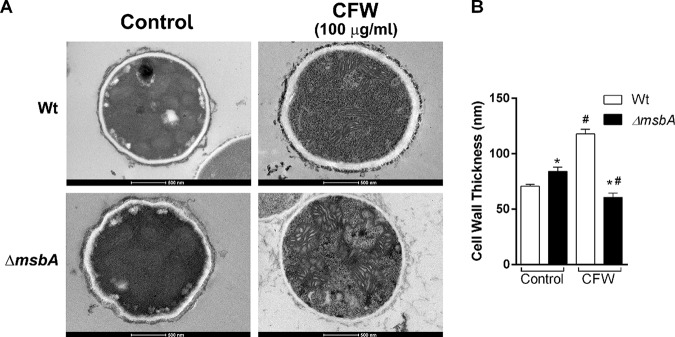
The *ΔmsbA* strain has altered cell wall thickness. (A) Transmission electronic microscopy images of hyphae from wild-type and *ΔmsbA* strains grown in liquid MM with or without CFW (100 μg/ml). Bars, 500 nm. (B) Cell wall thickness was quantified under each condition. Values are shown as mean ± SEM from 50 different sections. Asterisk represents statistical difference (*P* < 0.05) compared with wild type under the same growth condition. Number sign represents statistical difference (*P* < 0.05) compared within the group (wild type × wild type/*ΔmsbA* strain × *ΔmsbA* strain) under control condition.

In order to explore the evidence regarding the function of *msbA* in cell plasticity, we investigated the cell wall ultrastructure in the mutant strain by transmission electron microscopy (TEM). Interestingly, the *ΔmsbA* hypha cell walls were significantly thicker than the wild type under control conditions. In contrast, while wild-type cells showed an increase in cell wall thickness after CFW challenge, the mutant strain had a decrease in cell wall thickness compared to control condition (Fig. 5A). Comparing the two strains exposed to CFW, we observed that the mutant strain decreased about 50% in thickness (Fig. 5B). Taken together, these results suggest that *msbA* plays an important role in cell wall organization and plasticity that affects the fungal response to cell wall damage.

### A. fumigatus *msbA* modulates the expression of the CWI pathway genes.

**FIG 6:**
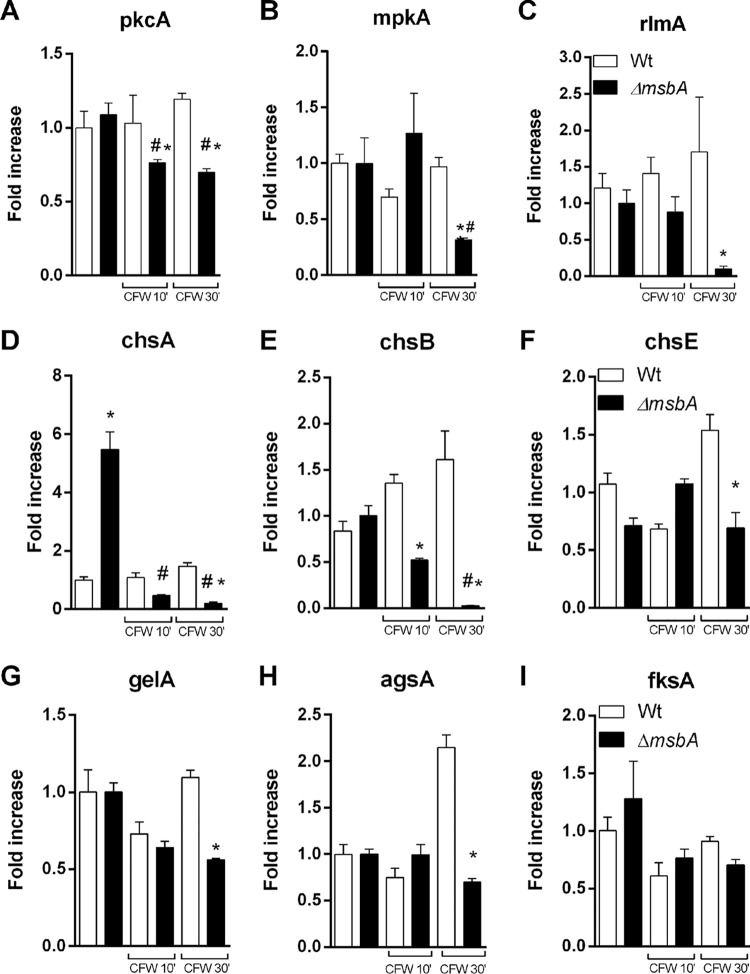
The *ΔmsbA* strain has decreased levels of expression of cell wall integrity pathway genes. Strains were grown in YG medium for 24 h at 37°C and treated with CFW (100 μg/ml) for 10 and 30 min. The control group was not treated with CFW. Fold increase in each strain represents the normalized mRNA abundance relative to the wild-type strain under the control condition. Values are shown as mean ± SEM. Asterisk represents statistical difference (*P* < 0.05) from wild type under the same growth condition. Number sign represents statistical difference (*P* < 0.05) compared within the group (wild type × wild type/*ΔmsbA* strain × *ΔmsbA* strain) under the control condition.

All the phenotypes mentioned above suggest an important role of *msbA* in the maintenance of the integrity of A. fumigatus cell wall, which may be reflected in the perturbed downstream signaling cascade due to abnormal sensing of cell wall disruption. To address this question, we evaluated the expression profile of genes related to cell wall biosynthesis and remodeling, such as the kinases *pkcA* and *mpkA* and the CWI transcription factor *rlmA*. Our results demonstrate that upon CFW exposition, the CWI signaling genes were downregulated in the mutant strain, while we were able to identify a slight upregulation of these genes in the wild-type strain (Fig. 6A to C).

We also examined the transcriptional levels of the CWI target genes such as chitin synthases (*chsA, chsB,* and *chsE*), α-1,3-glucan synthase (*agsA*), β-1,3-glucan synthase (*fksA*), and 1-3-β-glucanosyltransferase (*gelA*). Likewise, we observed lower expression levels of the evaluated genes after 30 min of CFW stimuli in the mutant strain, except for *fksA*. Curiously, the mutant strain expressed about five times more *chsA* mRNA than the wild type under the control condition (Fig. 6D to I). This finding is consistent with the cell wall alterations observed in the mutant and may help to explain phenotypes such as those described in Fig. 3. Our results demonstrate that the mucin MsbA regulates the CWI pathway under stress conditions, acting as a stress sensor.

### *msbA* contributes to downmodulating inflammatory responses after A. fumigatus infection.

**FIG 7:**
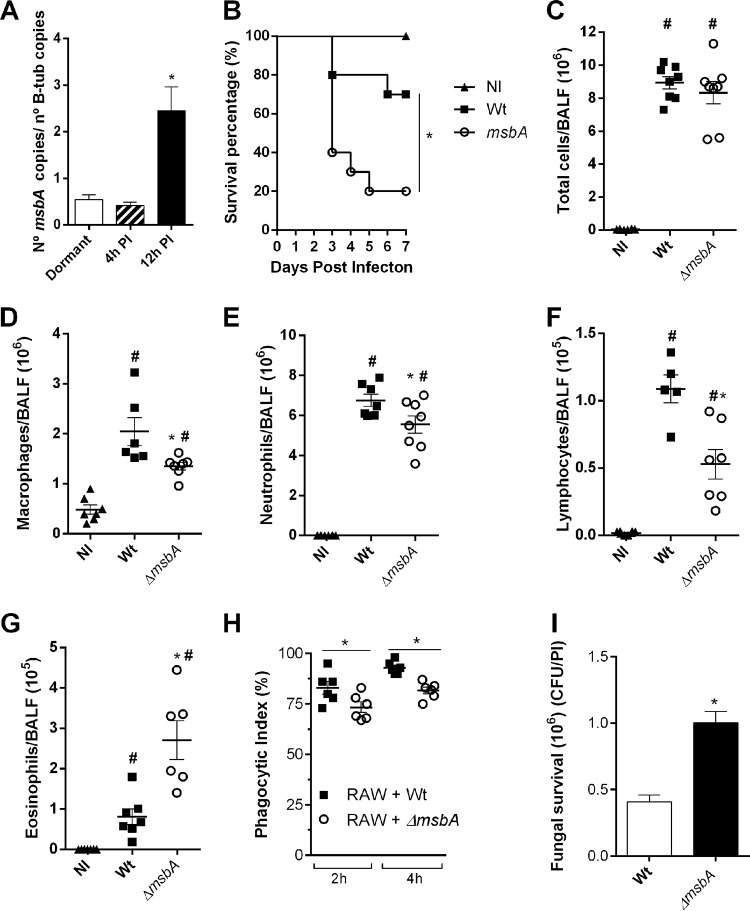
MsbA contributes to downmodulating the inflammatory response after A. fumigatus infection. (A) *msbA* mRNA expression during infection. A. fumigatus cells were harvested from lungs of infected animals after 4 h and 12 h (PI, postinfection). mRNA was extracted for qRT-PCR assay. Data are presented as mean ± SEM (*n* = 3 to 4 mice per group). *, significantly different (*P* < 0.05). (B) Lethality of mice infected with wild-type or *ΔmsbA* strain. Mice were infected intranasally with 40 μl of suspension containing 1 × 10^8^ conidia of wild-type or *ΔmsbA* strain, and mortality was monitored for 7 days. NI, noninfected. (C to G) Inflammatory infiltrate was analyzed in BALF of infected animals. Infection with *ΔmsbA* strain altered inflammatory cells recruitment into airways. Intranasally infected mice had BALFs harvested at day 1 postinfection for inflammatory cell infiltrate determination. Total cell (C), macrophage (D), neutrophil (E), lymphocyte (F), and eosinophil (G) absolute counts in BALF. Data are presented as mean ± SEM (*n* = 5 to 8 mice per group). *, significantly different (*P* < 0.05) compared to wild-type-infected group. #, significantly different (*P* < 0.05) compared to noninfected group. (H) *In vitro* phagocytosis was performed with RAW 264.7 immortalized macrophages challenged with wild-type or *ΔmsbA* strain for 2 h and 4 h for phagocytosis. The relationship between the number of macrophages containing conidia inside and the total number of macrophages was used to calculate phagocytic index. One hundred macrophages were counted for each coverslip. (I) Fungal survival was quantified as CFU/phagocytic index (PI). *ΔmsbA* conidium number after 6 h of phagocytosis was higher. *, significantly different (*P* < 0.05) compared to wild type.

Then, we evaluated whether *msbA* deletion affected A. fumigatus virulence after infection with a single inoculum of A. fumigatus in C57BL/6 immunocompetent mice. During the infection in mice, the expression of *msbA* was analyzed from fungal cells retrieved from the lungs 4 h and 12 h postinfection. Results show that, at the initial steps of fungal infection, expression of *msbA* was unaltered compared to the basal levels of dormant conidia. In contrast, 12 h postinfection, *msbA* was highly expressed, suggesting that it plays an important role in the vegetative growth of the fungus upon contact with host cells (Fig. 7A).

Survival rates of animals infected with the wild-type and knockout strains, after 7 days, showed that mice infected with the *ΔmsbA* strain were highly susceptible to the infection. Indeed, 80% of the mice infected with A. fumigatus *ΔmsbA* succumbed to infection at day 5 compared to only 30% of the ones infected with the wild-type fungus (Fig. 7B). To understand the underlying mechanism of this difference in mortality, we analyzed the cellular inflammatory profile in the airways. The results demonstrate the same amounts of total cells infiltrated into the alveoli as assessed from bronchoalveolar lavage fluid (BALF) of mouse groups infected with both strains 24 h postinfection (Fig. 7C). However, the profile of recruitment of cells was altered. The mutant strain infection induced the migration of a decreased number of macrophages, lymphocytes, and neutrophils (Fig. 7D to F) and of a higher number of eosinophils (Fig. 7G) recruited to the site of infection. In the lung tissue, there was a greater accumulation of eosinophils and neutrophils in mice infected with the *ΔmsbA* strain. The recruitment of macrophages presented no differences in the lung tissue between the two groups (see Fig. S2 in the supplemental material). We hypothesized whether these alterations in the immune system modulation would be the result of an alteration in the capacity of recognition of the fungus and activation of a proper response by phagocytes. In this way, *in vitro* phagocytic and clearance abilities of macrophages were accessed. After a 2-h assay, the wild-type strain was highly recognized and phagocytosed while the *ΔmsbA* strain showed a lower phagocytic index. The same pattern was observed after a 4-h assay; approximately 15% fewer conidia of the mutant strain were phagocytosed compared to the wild-type counterpart (Fig. 7H). Moreover, fungal clearance was accessed via CFU counting, after 6 h of incubation. It showed a significant increase in *ΔmsbA* conidium survival (Fig. 7I), meaning that macrophages were not able to clear *ΔmsbA* conidia properly. Thus, these results demonstrate that alterations of the cell wall remodeling mechanisms orchestrated by the stress sensor, MsbA, in A. fumigatus led to an altered recognition of conidia by phagocytes. Furthermore, beside recognition, the mutant strain is capable of evading killing machinery during phagocytosis and surviving more than the wild-type strain.

10.1128/mSphere.00350-19.2 FIG S2 Inflammatory response after A. fumigatus *ΔmsbA* infection altered inflammatory cell recruitment within the lungs. Mice were infected intranasally with 40 μl of suspension containing 1 × 10^8^ conidia of A. fumigatus wild-type or *ΔmsbA* strain. Lungs were harvested at 1 day postinfection for NAG (C), MPO (B), and EPO (A) determination. Data are presented as mean ± SEM (*n* = 5 to 8 mice per group). *, significantly different (*P* < 0.05) comparing *ΔmsbA* strain infection to wild-type infection. #, significantly different (*P* < 0.05) from noninfected (NI) group of mice. Download FIG S2, TIF file, 2.3 MB. Copyright © 2019 Gurgel et al. 2019 Gurgel et al. This content is distributed under the terms of the Creative Commons Attribution 4.0 International license.

**FIG 8:**
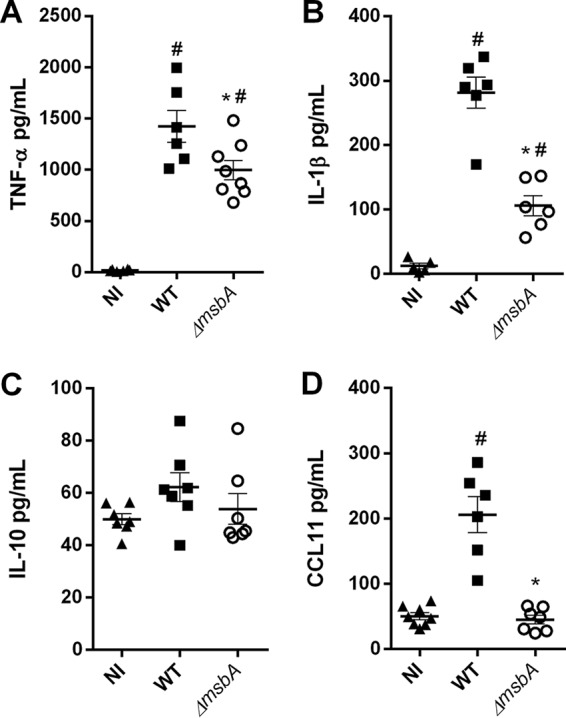
The *ΔmsbA* strain stimulates fewer inflammatory mediators in a model of A. fumigatus acute lung infection. Mice were infected with wild-type or *ΔmsbA* strains, and inflammatory mediators were analyzed by ELISA in BALFs at day 1 postinfection: TNF-α (A), IL-1β (B), IL-10 (C), and CCL11/eotaxin (D). *, significantly different (*P* < 0.05) from wild-type-infected group. #, significantly different (*P* < 0.05) from noninfected (NI) group.

The inflammatory response is a complex process that involves inflammatory signals such as cytokine and chemokine production (19). Therefore, we evaluated the role of the *ΔmsbA* mutant in the stimulation of immune system and the production of key mediators in BALF after A. fumigatus infection. Our data demonstrate that mice infected with the *ΔmsbA* strain showed a reduction of important inflammatory mediators like TNF-α and IL-1β, resulting in almost a 30% reduction of both cytokines, after 24 h of infection (Fig. 8A and B). The levels of IL-10 remained unaltered in the airways of infected animals compared to the noninfected mice (Fig. 8C). Curiously, despite the higher levels of eosinophils quantified at the infection site, CCL11/eotaxin1, one of the main chemokines responsible for recruitment of these cells, was detected at a lower concentration in BALF of *ΔmsbA* strain-infected animals (Fig. 8D). Taken together, these results demonstrate that, during infection, MsbA is an important signaling protein in A. fumigatus and takes part in fungal physiological processes that culminate in the recognition of the pathogen during lung infection in mammalian hosts. This evidence is especially important to guide cell infiltration into the airways and for the production of inflammatory mediators. The *msbA* gene is expressed at higher levels during the course of infection, which results in alterations that occur upon the loss of function of this mucin, modulating the overall innate immune response to affect the outcome of infection.

## DISCUSSION

In this study, we characterized the mucin-like protein MsbA in the human pathogen A. fumigatus. Here, we show that MsbA controls fungal development, as a result of the modulation of the cell wall integrity pathway, which culminates in the regulation of cell adhesion and biofilm formation and responses to cell wall stressors and to antifungal drugs. Moreover, MsbA deletion compromises the A. fumigatus recognition by phagocytes.

MsbA is involved in the vegetative growth of many yeasts and filamentous fungi. In C. albicans, Msb2 regulates the yeast filamentous growth by controlling Cek1 MAPK phosphorylation (8). Additionally, Brown et al. (11) demonstrated that in A. nidulans, MsbA regulates not only vegetative growth but also the conidiation process. Our data corroborate these results in A. fumigatus and highlight that *msbA* is required for the normal hyphal growth and conidiation of the fungus.

The fungal cell wall has a crucial role in pathogenic fungi as it is responsible for the pathogen survival, adaptation, and signaling under stressful conditions during infection (1, 20). It has been shown that Msb2 homologues in C. albicans, A. nidulans, and F. oxysporum regulate the response to cell wall stressors such as in the presence of Congo red, calcofluor white, and caspofungin (7, 8, 11). Brown et al. (11) showed that MsbA modulates the cell wall integrity (CWI) pathway, interfering with the production of chitin in the cell wall. The CWI pathway is the main signaling pathway controlling the synthesis of cell wall components in response to environmental stresses in S. cerevisiae (21). Here, we show that deletion of MsbA interferes in the expression of the A. fumigatus CWI pathway. Consequently, CWI target genes, such as the chitin synthase genes, were also misregulated, thus resulting in changes in the fungal cell wall morphometry. Collectively, our results corroborate literature described for different fungal species, showing that MsbA is similarly responsible for controlling the response of A. fumigatus in the presence of cell wall stress.

Changes in the cell wall composition can be responsible for altered recognition of the pathogen by host cells. This process can change the course of the inflammatory response, affecting the secretion of cytokines and chemokines and cell influx. Indeed, our results demonstrate an impairment of inflammatory cell migration into the airways after infection with the Δ*msbA* strain. These results were in accordance with the sensitivity assays and the expression levels of CWI genes. Along with the diminished influx of macrophages, lymphocytes, and neutrophils into the airways, there were reduced levels of TNF-α and IL-1β in mice infected with the *ΔmsbA* strain. This evidence indicates that the mucin MsbA is involved in the regulation of CWI in A. fumigatus, controlling the composition of fungal cell wall components (pathogen-associated molecular patterns [PAMPs]).

After infection, A. fumigatus cells are recognized by the immune system, triggering a series of events that promote clearance of the fungus from infected host tissues. This recognition involves primarily activation of pattern recognition receptors (PRRs) by the cell wall PAMPs (19). Alveolar macrophages are crucial components of the host defense against A. fumigatus infections not only because they promote the phagocytosis and killing of these fungal conidia but also because they initiate a proinflammatory response that recruits other leukocytes to the infection site, such as neutrophils (19, 22–24).

A number of factors, including inflammatory mediators such as cytokines and chemokines, mediate leukocyte influx into the site of infection. TNF-α is mainly secreted by alveolar macrophages upon stimulation, increasing the phagocytic activity of macrophages and neutrophil fungicidal activities against hyphae (25, 26). Besides TNF-α, alveolar macrophages secret other proinflammatory mediators, such as IL-1β and IL-1α (26). Werner et al. (26) showed a correlation between a decreased production of these cytokines and nonresponsive macrophages in A. fumigatus.

Altogether, our results show an important role of the A. fumigatus mucin MsbA in the fungal growth, maintenance, and plasticity of the cell wall. MsbA also contributes to fungal cell wall properties such as adhesion and biofilm formation and resistance to antifungals. Proper plasticity of the cell wall is crucial for fungal survival to phagocytosis and for the adequate innate immune response during mammalian pulmonary infection. Overall, our results show that the inflammatory profile triggered by infection with the A. fumigatus strain lacking mucin MsbA affects not only the host cell recruitment but also the production of inflammatory mediators. Additional studies are required to elucidate how this protein connects with the CWI pathway and the mechanisms whereby MsbA modulates the host response to A. fumigatus.

## MATERIALS AND METHODS

### Mutant construction.

Construction of the deletion cassette by *in vivo* recombination in S. cerevisiae was performed as previously described by Colot et al. (27). Briefly, fragments 5′ untranslated region (UTR) (1,505 bp) and 3′ UTR (1,532 bp) of the *msbA* gene (AFUA_4G04070) from the *ΔakuB^ku80^* strain (17) were amplified by PCR, as well as the gene *pyrG* from PCDA21. All fragments contained flanking sequences. The three fragments plus BamHI-EcoRI-cut pRS426 were transformed in S. cerevisiae (28). Positive transformant genomic DNA was transformed in Escherichia coli chemically competent DH5ɑ cells, aiming at amplification of the deletion cassette. The constructed deletion cassette was transformed in the wild-type Aspergillus fumigatus (*ΔakuB^ku80^*) through double homologous recombination, mediated by the polyethylene glycol-mediated protoplast technique (29). The Southern blot technique was utilized to confirm mutant construction.

To complement the Δ*msbA* strain, the cassette containing the *msbA* gene plus the two 1.5-kb flanking regions was PCR amplified using the genomic DNA from the *ΔakuB^ku80^* strain as a template (see Table S1 in the supplemental material). Protoplasts from the Δ*msbA* strain were transformed with the 5,736-bp PCR product and plated onto medium containing 200 μg/ml of CR and 100 μg/ml of CFW. Two revertants, which were able to grow under these conditions, were further analyzed by PCR, with the primer sets *msbA* FWD and *msbA* REV (Fig. S1A). The complemented strains were also tested for complementing phenotypes, and they yielded the same results (Fig. S1B to F). These strains were named Δ*msbA*::*msbA* 1 and Δ*msbA*::*msbA* 2.

10.1128/mSphere.00350-19.3 TABLE S1 Oligonucleotide sequences utilized for PCRs and qRT-PCRs. Download Table S1, PDF file, 0.03 MB. Copyright © 2019 Gurgel et al. 2019 Gurgel et al. This content is distributed under the terms of the Creative Commons Attribution 4.0 International license.

### Culture conditions.

A. fumigatus strains were grown in minimal medium (MM), complete medium (YAG), YG, or RPMI 2× agar, 2% (wt/vol) (30), at 28°C, 30°C, 37°C, or 42°C. When necessary, YAG TOP agar (1% agar) was utilized. Conidia were harvested and collected from agar medium after 48 h or 72 h. Next, conidia were diluted and counted in a Neubauer chamber. Conidiation (microcultivation) assay was carried out as described by Lin and Momany (31) with adaptations. Briefly, coverslips were placed on top of potato dextrose agar, on which conidia of both strains were previously deposited, and cultivated at 28°C for 48 h/72 h. For conidiophore structure observation, coverslips with aerial hyphae and conidiophores attached were stained using lactophenol blue solution, mounted on coverslips, and observed microscopically using ×40 magnification.

### Biofilm formation and adhesion assay.

A. fumigatus biofilm was produced and quantified according to the method of Mowat et al. (32) and Gravelat et al. (33), with adaptations. Briefly, 2 × 10^4^ conidia of each strain were inoculated in 200 ml MM into a flat-bottom 96-well polystyrene plate. The plate was incubated at 37°C for 24 h. After incubation, MM was removed from wells, and cells were washed four times with 1× PBS. For biofilm quantification, 150 μl of 0.5% (wt/vol) crystal violet was added to each well for 5 min, at environmental temperature. Mycelia were washed with sterile Milli-Q water. Remaining crystal violet was eluted with 200 μl 100% (vol/vol) ethanol per well. Ethanol solution was transferred to a new 96-well plate. Absorbance was determined at 570 nm.

The adhesion assay was performed according to the work of Shopova et al. (34), with adaptations. Briefly, conidium adhesion to the polystyrene plate surface was quantified through visual counting using inverted light microscopy. Conidia of each strain (5 × 10^5^) were diluted in 5 ml of RPMI. After 4 h of incubation at 37°C, RPMI was washed with 10 ml of sterile 1× PBS. The number of adhered conidia was determined in at least 6 optical fields and expressed as total percentage of conidia in each assay.

### Polarization.

Conidia of each strain (1 × 10^5^) were incubated at 37°C for 5 h in 5 ml of YG containing 50 mM hydroxyurea (HU), followed by washing with distilled water and then incubation in YG at 37°C. Samples were taken at 0 h, 0.5 h, 1 h, 1.5 h, and 2 h after HU blockage release, and germ tubes were quantified by direct counting of at least 100 cells per plate.

### Susceptibility assay for antifungal, cell wall, and osmotic stressor agents.

The antifungal Etest assay was conducted according to the method of Rocha et al. (3) with adaptations. Briefly, 1 × 10^5^ conidia in 100 μl 1× PBS were mixed into 10 ml RPMI TOP agar, placed over 15 ml RPMI agar in 90-mm plates, and left to dry. Etest strips were applied on the top of the plates, incubated at 37°C for 36 h, and photographed. MICs were determined as the minimum drug concentration of the inhibitory halo intercepting the test strip. To evaluate growth under cell wall and osmotic stress, concentrations of Congo red (200 μg/ml), calcofluor white (50 μg/ml), and sodium chloride (400 mM) were used. Five-microliter drops with 10× dilutions of both strains were plated in MM plus the stressor agent and grown for 30 h at 37°C. To evaluate sensitivity to nikkomycin Z, 1 × 10^4^ conidia were cultivated in MM in a 24-well plate containing serial dilutions of the drug (50 mM, 100 mM, and 150 mM) at 37°C for 48 h and photographed.

### TEM analysis.

Wild-type and Δ*msbA* strains (1 × 10^7^ conidia) were grown in liquid MM for 24 h at 37°C prior to exposure to CFW (100 μg/ml) for 2 h. Cells were processed essentially as described previously with modifications (35). Briefly, mycelium was fixed in 0.1 M sodium phosphate buffer (pH 7.4)-2.5% (vol/vol) glutaraldehyde for 24 h at 4°C. Samples were encapsulated in 2% (wt/vol) agar and subjected to fixation (1% OsO_4_), contrast (1% uranyl acetate), ethanol dehydration, and a two-step infiltration process with propylene oxide-EMbed 812 (Electron Microscopy Sciences) of 16 h and 3 h at room temperature. Additional infiltration was provided under vacuum at room temperature before embedding in BEEM capsules (Electron Microscopy Sciences) and polymerization at 60°C for 72 h. Semithin (0.5-mm) survey sections were stained with toluidine blue to identify the areas of greatest cell density. Ultrathin sections (60 nm) were prepared and stained with uranyl acetate (1%) and lead citrate (2%). Transmission electron microscopy (TEM) images were obtained using a Tecnai G2-12-SpiritBiotwin FEI electron microscope at an acceleration voltage of 120 kV (Center of Microscopy from UFMG, Brazil) using a charge-coupled device (CCD) camera. Cell wall thicknesses of 50 sections of different germlings were measured using magnification of ×26,500 and ImageJ software analysis (13).

### RNA extraction and real-time qRT-PCR procedures.

Mycelia were disrupted by grinding in liquid nitrogen with a pestle and mortar. Total RNA was extracted using TRIzol (ThermoFisher Scientific) according to the manufacturer’s protocol. Samples were treated with DNase I (Invitrogen). RNA concentration and quality were assessed with a nanophotometer (NanoDrop; ThermoFisher Scientific). A total of 2 μg of DNase-treated total RNA from each A. fumigatus strain was reverse transcribed using the SuperScript III reverse transcriptase kit (Invitrogen) and oligo(dT) primers. Real-time RT-PCR was conducted using Power Sybr green PCR master mix (Applied Biosystems). Primers used are listed in Table S1 in the supplemental material. RT-PCR was performed in duplicate in a 7500 Fast real-time PCR system (Applied Biosystems) according to the manufacturer’s instructions. Nontemplate controls (NTC) were used to confirm elimination of contaminating DNA in every run. After completing PCR, melt curve analysis was performed to confirm the absence of nonspecific amplification products. The results were normalized using β-tubulin copy number, calculated in reference to a standard curve with known amounts of A. fumigatus genomic DNA (36).

### Fungicidal clearance capacity by phagocytic cells.

*In vitro* phagocytosis was performed according to the method of Bom et al. (35) with adaptations. Immortalized macrophages (1 × 10^5^; RAW 264.7 strain) were cultured in 1 ml of Dulbecco’s minimal essential medium (DMEM), supplemented with 2.5% (vol/vol) fetal bovine serum (FBS) in a 24-well plate containing 13-mm round coverslips. Cells were maintained at 37°C in a 5% CO_2_ atmosphere. After incubation, wells were washed 3 times with DMEM with no antibiotics. One milliliter of DMEM plus 2% (vol/vol) FBS plus 1 × 10^6^ conidia of both strains (1:10) were added to each well. Samples were incubated for 2 h and 4 h. Each well was washed 4 times with 1× PBS at 37°C. The 13-mm round coverslips were stained with the Panoptic kit (Laborclin) and submitted to phagocytosis counting. For fungicidal clearance evaluation, 1 × 10^6^ RAW macrophages were incubated in DMEM-2.5% (vol/vol) FBS in a 24-well plate. Wells were washed prior to incubation with DMEM + 2% (vol/vol) FBS plus 1 × 10^7^ conidia of both strains (1:10) in each well. Samples were incubated at 37°C in a 5% CO_2_ atmosphere for 6 h. Wells were washed for cell lysis with sterile water, and the remaining conidia were plated in YAG medium to quantify the CFU.

### Ethics statement.

Animal experiments were approved by the Institution Ethics Committee (Comissão de Ética no Uso de Animais, CEUA/UFMG, protocol number 187/2018), according to Brazilian national guidelines on animal work (Conselho Nacional de Controle de Experimentação Animal [CONCEA]).

### Animal infection and *in vivo* assays.

In this study, we used male and female 8- to 12-week-old C57BL/6J specific-pathogen-free (SPF) mice. Prior to infection, mice were anesthetized by inhaling up to 3% isoflurane (Biochimico, Brazil) with oxygen. Mice were intranasally infected with 1 × 10^8^ conidia of the A. fumigatus wild-type or *ΔmsbA* strain in 40 μl of sterile PBS. After 24 h of incubation, infected mice were euthanized with a solution of 180 mg/kg of body weight of ketamine and 24 mg/kg of xylazine. Subsequently, bronchoalveolar lavage fluid (BALF) was harvested by washing the lungs twice with 2 ml of PBS. Fluid was centrifuged, and cell pellets were used for total and differential leukocytes counts. Supernatants were used for cytokine and chemokine quantification (37). Prior to removal and freezing of the right lobes of the lungs for myeloperoxidase (MPO), *N*-acetylglucosaminidase (NAG), and eosinophil peroxidase (EPO) analysis, lungs were perfused with 5 ml of 1× PBS (38–40). Additionally, lungs were harvested for the fungal burden analysis.

### Cytokine and chemokine measurement.

DuoSet ELISA kits (R&D) were used to quantify cytokine and chemokine levels in BALF, according to the manufacturer’s instructions.

### Statistical analysis.

Experiments were performed at least twice. Data are presented as the mean ± SEM. Statistical differences were analyzed with one-way analysis of variance (ANOVA), followed by Holm-Sidak posttest. Normal distribution was evaluated by the D’Agostino-Person test. Nonparametric data were analyzed with the Kruskal-Wallis test. Mann-Whitney test (nonparametric) or *t* test (parametric) was used to compare two groups. Survival analysis was performed using log rank test. Statistical significance was set as *P* < 0.05. Graphs and analysis were performed using GraphPad Prism 6.0 software (GraphPad Software Inc., San Diego, CA, USA).
