# Supplementary material for: The Aspergillus fumigatus Mucin MsbA Regulates the Cell Wall Integrity Pathway and Controls Recognition of the Fungus by the Immune System
Source: mSphere. 2019 Jun 19;4(3):e00350-19. doi: 10.1128/mSphere.00350-19 (PMC6584374; doi:10.1128/mSphere.00350-19)
Supplement: TABLE S1 [file mSphere.00350-19-st001.pdf]

**qRT-PCR Oligonucleotides**	**Sequence**
Msb2A 5’ Fwd	5’-GTAACGCCAGGGTTTTCCCAGTCACGACGACTATATCATTAGGTAGGC-3’
Msb2A 5’ Rev	5’-GTGCCTCCTCTCAGACAGAATGGTTTTGATACCAAAGCGACTGC-3’
Msb2A 3’ Fwd	5’-GAGCATTGTTTGAGGCGAATTCCTGATAAACTGAACCCTACGATG -3’
Msb2A 3’ Rev	5’-GCGGTTAACAATTTCTCTCTGGAAACAGCCATCGCAAGCATGGGGCTG-3’
PyrG Fwd	5’-ATTCTGTCTGAGAGGAGGCAC-3’
PyrG Rev	5'-GAATTCGCCTCAAACAATGCTC-3'
**qRT-PCR Oligonucleotides**	**Sequence**
Afu pkcA Fwd	5’-CCGAAGTTCTGTTGGCTCTC-3’
Afu pkcA Rev	5’-CAGAGACCGTAATCGGCAAT-3’
Afu mpkA Fwd	5’- GGCCATCAAGAAGGTTACCA -3’
Afu mpkA Rev	5’- TGAAATTGTCTGGTCGTGGA-3’
Afu rlmA Fwd	5’-GACGCCGATCTCTGCTCTAC-3’
Afu rlmA Rev	5’-GGAGTGGGGAAGGTTAGAGG-3’
Afu chsA Fwd	5’-CTGGAGTGTGGCTGGTCTCT-3’
Afu cshA Rev	5’-GCGTGTGAAAGCAGTATGGA-3’
Afu chsB Fwd	5’-GCTCTCCACTGTCGGTCTCT-3’
Afu chsB Rev	5’-GGTCGTTGTTGATGGTGTTG-3’
Afu chsC Fwd	5’-TTGCTGCGAGTTTGTATTCC-3’
Afu chsC Rev	5’-GCCAGTAGGATGCCAAAGAG-3’
Afu chsE Fwd	5’-TGGTGTTCGTTGACTTGCTC-3’
Afu chsE Rev	5’-TCATCCATCCAACCATTTCC-3’
Afu agsA Fwd	5’-CCAACACCTGGAAGATGACC-3’
Afu agsA Rev	5’-AACACCGACCGATAGAAGGA-3’
Afu fksA Fwd	5’-AAGCAATCGAAGCTCAGGAA-3’
Afu fksA Rev	5’-ACCAATCCCATAGAGCGAAC-3’
Afu gelA Fwd	5’-CACTGGCTACGGTCTTCCTC-3’
Afu gelA Rev	5’-CATTGTTGCCGCTAATCTCC-3’
Afu msb2A Fwd	5’- ACTCTCTACAACGGAGCAAAC -3’
Afu msb2A Rev	5’- GTGGTAGTTTCGGGTGGATAAG -3’
